# Clinical Genetics and the Practitioner

**Published:** 1960-10

**Authors:** Thomas E. Oppé

**Affiliations:** Lecturer in Child Health, University of Bristol


### CLINICAL GENETICS AND THE PRACTITIONER

BY

*THOMAS E. OPPÉ

*Lecturer in Child Health, University of Bristol*

During the past few years there has been a remarkable increase in interest concerning human genetics. Undoubtedly the main reason for this has been the advances in knowledge that have come at a time when the demand for a new knowledge has been urgent and articulate. The nineteenth and early twentieth centuries will go down in history as the golden age of scientific medicine, marked by an intense pre-occupation with the external causes of disease. Bacteriology disclosed the bacterial and viral agents causing infective disease, nutritional science revealed the role of the vitamins and other nutritional elements in the causation of deprivation syndromes. Cancer research was much occupied with chronic irritants from spindle-oil to smegma, and "imferon" to cigarettes.

The result was a depersonalized, scientific medicine which elucidated most of the diseases due to infection, malnutrition and other external agents, leaving the degenerative diseases and neoplasia as a hard and unsolved core. The science was good, the depersonalization was bad. The individual, encouraged by the psychiatrists, the geneticists and the Welfare State, has risen again to assert his rights. Why do some smokers get cancer and others not, why do only a minority of children who have had a streptococcal infection get rheumatic fever or acute glomerular nephritis, why do some slothful, gluttonous business executives have coronary artery disease while others equally indolent and gluttonous do not? Peering at Aschoff nodes, or thrombosed coronaries, or meticulously classifying cells in tumours of the bronchus are unlikely to answer the questions. The geneticist, however, may well have something to contribute.

The year 1945 saw the birth of the atomic age which may lead us to a fearful and obvious calamity; however, even if we escape the full horror of atomic war, there are more subtle ways in which nuclear radiation may be harmful. Ionizing radiations distort and damage dividing cells with hazardous implications for the heredity of man. These dangers have brought into being the new science of radiobiology. This is of immense concern to the geneticist and implicates every doctor who sends a patient for a radiographic investigation.

Just as our new concern is with the essential individuality of our patients, so it is associated with a new outlook towards the essential sameness of the family of mankind. Policies of repression and inequality based upon the alleged genetic inferiorities of Jews or Negroes are being rightly criticised. Advances in genetic knowledge can give us cold, objective facts with which to sweep away emotional prejudices and misconceptions.

There is now almost no field of human activity in which the geneticist is not involved. Even such moral and religious issues as artificial insemination (A.I.D.) have a genetic aspect. No-one would now pretend that the sought-for perfection of the human race can be brought about by eugenic means, or that policies of selective mating or discriminative sterilization would alter profoundly the destiny of man. However, human genetics can no longer be regarded as an amiable hobby pursued by lovers of rare syndromes, or a fanatical cause which promised Utopia in an imperfect world.

* Present address: Paediatric Unit, St. Mary's Hospital London, W.2.

2

Modern methods of communication, notably the television, have brought these questions and some of the answers into the vision of millions of people. Many of these people have a personal genetic problem for which they will turn to their family doctor for advice. In many cases the doctor will be able to provide wise and authoritative guidance, in others he may feel the need for expert help. The purpose of this article is to review some of the newer work in the field of human clinical genetics in the light of the human problems which have come to our notice at the Bristol Children's Hospital Genetic Clinic. In doing so our main aims have been to put the doctor in the genetic picture and, at the same time, indicate the areas where specialized knowledge may be helpful.

### HUMAN GENETIC PROBLEMS

An analysis of the genetic clinic material indicates clearly the type of problems for which patients seek genetic advice.

| | |
|---|---|
| Premarital problems | 4 |
| Whether to have children | 2 |
| Whether to have more children | 70 |
| Whether pregnancy should be terminated or sterilization is justifiable | 8 |
| Whether a condition will be transmitted by their children | 5 |
| Doctor or patient interest | 13 |
| Reasons not clear | 3 |
| | 105 |

In the majority of cases genetic advice is sought after the birth of an abnormal child. The abnormality may be known or merely suspected to be an inherited one, and the parents' concern is whether subsequent children may be abnormal. Rather fewer cases are seen because one or other partner in a marriage or projected marriage is cognisant of some familial disorder and wishes to know the genetic risks of having children. Others who have families containing both normal and abnormal individuals are concerned whether their normal offspring may transmit the disease to future generations. Although usually there is no urgency about genetic advice, sometimes an emergency arises.

**Mr. and Mrs. C. 45369**

Mr. C. was severely disabled with cerebellar ataxia which he had inherited from his mother. His only daughter was similarly affected and greatly handicapped. When Mrs. C. became pregnant again she was extremely worried that the foetus might be abnormal. As the inheritance is clearly dominant, advice was given that the chances were 1 : 2 of the child being affected. The anxiety of the mother faced with this poor genetic prognosis was sufficient to justify termination of the pregnancy and subsequent sterilization.

In other cases advice is sought because the family hope for an explanation for the puzzling abnormality, and in some because either the family or the doctor are interested in the genetic aspects of a condition.

The essential facts which the geneticist must have are (*a*) the diagnosis and (*b*) a complete pedigree of the family. It is unfair to expect the geneticist to make diagnoses of obscure cases from the parents' description. Therefore full clinical data are always appreciated concerning all suspected members of the family. Likewise, it is unfair to extract details of family trees often including illegitimacy, consanguinous marriages, etc., from patients unless they are prepared and co-operative. Before referring a case for genetic advice it is helpful if an accurate diagnosis is made, the problem is clearly defined and the parents are aware of the sort of questions they will be asked and the type of help that they may expect.

Most problems resolve themselves into three main groups:

I The disease in question is not inherited and genetic causes and transmission can be ruled out.

II The disease is inherited and its genetics follows a regular, known pattern of inheritance.

III The disease is familial, and genetic factors are believed to be involved. However, either through the number of genes involved or because of considerable environmental modification of gene influence, no regular pattern of inheritance is discernible.

Although the material at the genetic clinic is highly selected, it is of some interest to see the proportion of patients attending in each of these groups. It should be borne in mind that there is usually some special doubt or difficulty leading to referral in cases of Group I, the non-genetic group. Commonly, this is to provide expert reassurance. In Group II, the diseases definitely and regularly inherited, a good deal of appropriate counselling is done by general practitioners or consultants and there is no need for attendance at a special clinic. It is in Group III, where genetic mechanisms are involved in irregular or obscure fashion that most difficulty arises, and the help of the expert geneticist may be most useful. The genetic clinic does not represent a true cross-section of patients who might be grateful or benefit from genetic advice. A large section is adequately advised elsewhere.

GROUP I. CONDITIONS THOUGHT TO BE NON-GENETIC IN ORIGIN (10 cases)

Cerebral palsy probable due to perinatal trauma or anoxia is the most common in this group, and some cases of mental sub-normality may also be assigned here. Parents of children suffering from leukaemia and other neoplasms, aplastic anaemia and occasional other disorders have been reassured. The problems here is usually a condition which has multiple aetiologies, and separation between those largely caused by heredity and those due to enviromental causes is difficult.

**Mr. and Mrs. V. 60560**

Mr. and Mrs. V. are first cousins. Their first child was born after a difficult labour ending in traumatic forceps delivery. The child's condition at birth was poor and during the neonatal period he had signs of cerebral haemorrhage. At one year of age he was grossly retarded, small-headed and had a spastic quadriplegia. The diagnosis was cerebral palsy (spastic quadriplegia with mental retardation). The family history was completely negative, except for the consanguinity.

The problem was whether there were genetic risks for future pregnancies. Cerebral palsy may be genetic in origin in some cases, but there is a considerable body of evidence that many cases are caused by perinatal damage to the brain. In this case there was undoubted perinatal trauma, both mechanical and hypoxic. It seemed unnecessary to postulate a genetic cause in addition to an adequate environmental one. A reassuring genetic prognosis was given, and the parents were told that the disease in question was unlikely to be the result of an hereditary taint.

Some rare conditions are seen because their aetiology is quite unknown and the doctor wishes to know whether hereditary factors have been implicated.

**Mr. and Mrs. N. 64525**

This is a normal, healthy family, except for their third child who was born with all the manifestations of the Moebius syndrome. He had nuclear agenesis (paralyses of the left facial and external rectus nerves), micrognathos, stridor, absent pectoralis major muscles, absent left nipple, a deformed left hand and dextrocardia. He is making good progress, although he is generally retarded. They had subsequently another child who is quite normal. They were referred to the clinic partly because they were interested in establishing a reason for the malformation and also because of an underlying anxiety that their three normal children might transmit the disease.

Reference to recent literature on the subject showed that there is no obvious familial tendency in this condition, the parents were therefore reassured not only as far as future pregnancies of their own were concerned but also for their children's potential children.

Elimination of important genetic factors, which is the main problem in this group, rests on two main principles. Firstly, that the condition in question is known to have an external causation and the causation was operating in the particular case, and secondly that there is not satisfactory evidence of genetic participation in the condition in question. The latter postulate is difficult to apply. What is satisfactory evidence that genetic inheritance plays a part in the causation of a particular condition? Such evidence may be clinical, gemellological, or experimental.

Clinical evidence is convincing when a condition is clearly shown to be familial and the distribution of affected members of the family follows a regular pattern of Mendelian inheritance. More often it is merely suggestive in that a condition appears in more members of a family than would appear likely by chance. Caution is needed in interpreting such findings. A disease may run in a family because of infection, e.g. tuberculosis, or because of shared environment, e.g. pneumoconiosis in coal-mining families. Complex multi-factorial causative factors may be involved. There is a familial predisposition to coronary thrombosis. This might be due directly to a genetic disturbance in cholesterol metabolism or, much more indirectly, to the fact that over-conscientious, professional families tend to place their offspring in professional or academic careers, or that the eating habits of individuals usually follow by example the dietary habits of their parents.

The study of twinning (gemellology) is a valuable tool. Identical (uniovular) twins have a common genetic inheritance, dissimilar (binovular) twins are no more genetically similar than any sibs. If it can be clearly shown that a characteristic or condition is shown more frequently in identical twins than in dissimilar twins there is a reasonable assumption that the characteristic or condition is determined by heredity. It has recently been pointed out that twins form two per cent of any doctor's practice and there is a tremendous amount to be learned from them (Nance, 1959).

Experimental genetic evidence is difficult to evaluate. Man is unsuitable for genetic experiments owing to his unfortunate habits of marrying for love and producing rather few offspring over a long period of time. Animals or plants which can be selectively mated and produce many generations are more suitable. However, much caution has to be used in interpreting results gained by the study of other species.

Two new approaches to problems of identifying the genetic causation of disease or association with disease are yielding extremely interesting results. The first approach is that of blood group associations with susceptibility to disease. There is no doubt that the blood group of an individual is a genetically inherited characteristic. There are extremely rare exceptions of dissimilar twins sharing blood groups due to foetal inter-placental transfusion; however, these blood group chimeras are statistically negligible. If it can be shown that a disease is consistently associated with the possession of one or other blood group it is reasonable to assume that there may be some genetic factors in that disease. So far, blood group associations have been most firmly established with disorders of the upper alimentary tract, duodenal ulceration, gastric carcinoma and Addisonian (pernicious) anaemia (Roberts, 1959a). Further work in this field must be laborious and involve the statistical correlation of many thousands of individuals whose blood groups are known and who suffer from diseases suspected of being linked to blood groups.

Another recent and important advance is the direct demonstration of chromosome abnormalities in man. New methods of tissue culture and the staining of dividing cells made it possible for Tjio and Lefan in Sweden and Ford and Hamerton (1956) in Britain to identify and differentiate the chromosomes in man. The first result was to show that the normal number of chromosomes in human body cells was forty-six, not forty-eight as had previously been believed. Extension of this showed that some pathological conditions were clearly associated with abnormalities in the number of chromosomes. Such a demonstration is proof of a genetic contribution to the disease in question. A fuller discussion of the clinical value of this method will be given below.

GROUP II. CONDITIONS DEFINITELY AND REGULARLY INHERITED (46 cases)

(*a*) Those due to dominant genes: sixteen families (including hereditary spherocytosis, achondroplasia, cranio-facial dysostosis, muscular dystrophy (peroneal and facio-scapulo-humeral types), vitamin D resistant rickets).

(*b*) Those due to recessive genes: eighteen families (including fibrocystic disease of the pancreas, progressive infantile spinal muscular atrophy, gargoylism phenylketonuria).

(*c*) Sex-lined recessive: twelve families (predominantly the coagulation disorders, haemophilia and Christmas disease, and muscular dystrophy of pseudo-hypertrophic type).

Patients seek advice for these conditions, either pre-maritally, before having children, or when they have an affected child. Patients who have not yet had children may either suffer from an inherited disease themselves or be aware of members of the family who are afflicted. Again, it is important to know the diagnosis, to obtain a full family history and to be aware of the known facts concerning the transmission of the disease. Some typical problems have been:

**Miss J. D. 36487**

She was a student nurse aged 21. Her brother is a known haemophiliac attending the children's hospital. Her maternal grandfather and one of his brothers were thought to be "haemophiliacs". One of her great-grandmother's sisters had a male child who "bled easily". Miss J. D. was engaged to be married and wished to know whether any children she might have were likely to be "haemophiliac".

The diagnosis was clear, true haemophilia in her brother. The family tree was classically distributed for the sex-linked recessive inheritance of haemophilia, which made it most unlikely that her brother was haemophiliac because of a mutant gene. On this evidence alone the genetic prognosis is calculated as follows. It is almost certain that her mother is a carrier, therefore she herself has a fifty-fifty chance of also being one. If she is not, she need have no fears that her offspring will be affected. If she is, then statistically half her daughters will be carriers, and half her sons afflicted with the disease. The most important question is whether or not she is a carrier. In some cases the female carriers of haemophilia can be identified by estimating the level of anti-haemophilic globulin (A.H.G.) in the blood. However, this is by no means consistently lowered in carriers and in Miss J. D.'s case was normal. Follow-up has not been done, but it would be interesting to know whether or not she has limited her family because of the possibility of her being a carrier.

This example is a good one of the necessity for separating the genetic risks from the wider question of whether a couple are advised to have children or not, the latter problem includes moral, religious and financial issues. Some people believe sincerely that it is wrong to bring into the world children who may be malformed or diseased, others believe it is wrong to interfere with natural reproduction. The geneticist should be entirely objective and generally will give advice only on the genetic risks. However, as a medical practitioner, he may reasonably give further information of a medical nature. In this case, it is reasonable to point out that haemophilia is a disease of variable severity not necessarily incompatible with a normal life and also that advances in treatment may well occur in the near future which will make haemophilia less of a handicap.

**Miss C. 65428**

This 23-year-old unmarried girl had affronted her family by bearing the child of a U.S. Serviceman, with whom she was much in love. The baby showed unmistakable signs of being affected by progressive infantile spinal muscular atrophy and died when a few months old. The couple were seriously considering marriage, but both wanted children.

The condition is recognized as being due to an autosomal recessive gene. It was explained to the couple that if they married, the chances of further children being affected were one in four. On the other hand, if they did not marry each other but sought other partners, it was extremely unlikely that any of the offspring would be affected. The reason for this is that both must be 'carriers' of the rare gene for this condition and it would be improbable that such a choice of mating should occur again. It was later heard that they had attempted to solve this dilemma by attempting a further pregnancy, still unwed. Presumably if the second child were normal they would get married, if it should be affected they would "call it a day". This eminently practical and enterprising, if immoral, approach could hardly have been suggested by the clinic!

**Mr. and Mrs. W.** had normal family histories, but their first child died of progressive infantile spinal muscular atrophy. At the clinic it was explained that the chances of future children being affected were one in four. They decided that they would try again and a second child was born. She was also affected and died within a few months. They again attended the clinic having been advised elsewhere that it would be foolish to have another pregnancy. It was again explained that the odds were three to one in favour of having a normal child. With considerable courage they decided to have another child, but at the same time were put in touch with an Adoption agency. Their third child, unfortunately, was affected, but they were able to adopt a baby.

This case emphasies the importance of the parents deciding for themselves how the genetic risk should be interpreted. There is no doubt in this case that their decision to undertake further pregnancies was influenced by the knowledge that the disease was an almost invariably fatal one. It is perhaps natural that parents should accept greater genetic risks when the outcome is either normality or early death, rather than if there is a possibility of chronic handicapping illness in the child.

Most doctors will be familiar with the classical forms of Mendelian inheritance and it is with the conditions showing these regular patterns that the geneticist is on safe ground. Most of these conditions are due to the action of single genes. If the gene exerts such a powerful effect that possession of the gene in unit dose (heterozygous) produces a clinical abnormality we term that a 'dominant' gene. If, however, the gene only produces the clinical effect in double dose (homozygous) it is termed recessive. In the case of recessive genes in single 'dose' (heterozygotes) the presence of the gene may be detectable by special tests (Hsia, 1959). Until recently our knowledge of genes and their action has been based largely on conjecture, by studying their effects. There have, however, been some real advances in knowledge, both of the structure of genes and the ways in which they produce their effects.

The gene is a distinct but very small particle composed of a nucleic acid (D.N.A.) and proteins. The protein part is probably the component that is responsible for the unique ability of genes to transmit 'genetic' information. The protein part of the particle consists of polypeptide chains which are folded in a specific manner. Each polypeptide chain has a regular sequence of aminoacids along its length. Changes in a gene might then be due to alterations in the amino-acid sequence or to differences in folding of the polypeptide chains. It is only by the continued handing on to reproducing cells of these chemical structures that genetic inheritance can work. It is thus tempting and not too speculative to say that primary gene effects will be found by tracing abnormalities in protein structure. This hypothesis has received very convincing conformation and has opened up a vast new field of medicine, biochemical genetics.

The exact chemistry of proteins is only just being explored, and it is encouraging to know that much of the work is British. It is fortunate for medicine that the protein chemists have been interested in two proteins of much interest to us, insulin and haemoglobin. The work on haemoglobin is of greatest interest to geneticists. A series of diseases has long been known, characterized by chronic haemolytic anaemia and abnormalities of the red blood cells. These diseases, notably sickle-cell anaemia and thalassaemia, were also noted to be prevalent among certain races and families. The basic disorder in these diseases has been shown to be abnormalities in the haemoglobin molecule. Furthermore it is strongly suspected that the molecular differences are due to changes in the amino-acid sequence of the polypeptide chains. The situation is further complicated because it is known that there are two different polypeptide chains in the haemoglobin molecule, an $\alpha$ chain and a $\beta$ chain. This probably means that two genes are involved in the 'construction' of a single haemoglobin molecule. The additional evidence needed to confirm this concept is to demonstrate biochemical abnormalities in the genes corresponding to those that occur in the haemoglobin molecule. It is not yet possible to do this. At least twenty variants of human haemoglobin are known. Not all lead to clinical effects. An individual who inherits one abnormal haemoglobin gene will produce both normal and the aberrant haemoglobin. These heterozygotes are said to possess the "trait". It is interesting that there is some evidence that red cells containing sickle-cell haemoglobin (Hb–S) are resistant to malaria parasites. Therefore, individuals with the sickle-cell trait are more likely to survive naturally in malarial zones. This is sometimes cited as evidence that possession of one undesirable recessive gene may, in fact, be of biological advantage. Homozygotes for a haemoglobinopathy are likely to show the full manifestations of the disease with anaemia, splenomegaly and bone changes. Apart from the immense genetic interest of these conditions, they are of some clinical importance in Britain owing to the influx of immigrants of either negro or Mediterranean extraction.

The theory of a biochemical or structural abnormality in a gene altering the molecular structure of a body protein has also influenced the concept of "inborn errors of metabolism". These were familiar to most doctors through the famous Dalmatian coach hound. This animal shares with people suffering from an error of amino-acid metabolism the symptom of alkaptonuria. This is a harmless metabolic quirk. A similar inborn error of protein metabolism is by no means harmless, that is phenylketonuria. Phenyl-ketonuria is a familial disorder characterized by mental deficiency, hyperkinetic behaviour, dry eczematous skin, blonde hair, blue eyes and often epilepsy. Biochemically, there is an elevation of phenylalanine level in the blood and phenylpyruvic acid is found in the urine. The disease is of importance because it is one of the few causes of mental deficiency that are treatable. Treatment, however, to be effective must start early in life. The essential lesion (the inborn error of metabolism) is an inability of the affected individual to convert phenylalanine into tyrosine. This metabolic block is due to inactivity of the enzyme phenylalanine-hydroxylase. It is believed that the enzyme deficiency is determined by the presence of a double "dose" of recessive gene. A single "dose" of recessive gene produce a disturbance of phenylalanine metabolism detectable only by special tests.

If we schematically represent the reaction phenylalanine to tyrosine as, $A \xrightarrow{x} B$, where $A$ is phenylalanine, $B$ tryosine, and $x$ is phenylalanine-hydroxylase, we may represent the phenylketonuric patient as

$$\begin{matrix} A \\ A_1 \not\rightarrow b. \\ A_2 \end{matrix}$$

"$A$, $A_1$, $A_2$," indicate that there is a pile-up of phenylalanine, some fraction of which excess is metabolized abnormally, producing $A_1$ (phenylpyruvic acid) and $A_2$ (ortho-hydroxy-phenylacetic acid) which appear in the urine; and "$b$" indicates a lack of tyrosine and its further metabolites. Although the primary gene effect is exerted on only one enzyme, the clinical effects are widespread. The accumulation of abnormal metabolites leads to excretion of an abnormal urinary constituent, it is probably responsible also for the brain damage and epilepsy. Apart from the effects due to phenylalanine excess there are the sequelae of tyrosine depletion. These include lack of synthesis of melanin and adrenaline, so explaining the lack of of pigmentation and low blood adrenaline.

Phenylketonuria is treated by giving a diet deficient in phenylalanine; this spares the body from excess and the toxic effects therefrom. The diet is expensive, unpalatable, and difficult to control, nevertheless considerable success has been claimed. It is useless if treatment is not started early. Early diagnosis is the best way of getting early treatment and it is obvious that one cannot wait until there are signs of mental retardation. Unfortunately, the disorder cannot be detected at birth. During the early days of life there may be an elevation of plasma phenylalanine, next ortho-hydroxy-phenylacetic acid appears in the urine and only after 3 to 6 weeks does the easily identified phenylpyruvic acid appear. Phenylpyruvic acid is tested for either with (1) ferric chloride or (2) "phenistix" (Ames & Co.).

(1) A few drops of ten per cent ferric chloride solution are added to a specimen of urine, previously acidified with a few drops of HCl. If phenylpyruvic acid is present an intense green colour results.

(2) The end of the test strip is dipped in urine or moistened by pressing against a damp napkin. After 30 seconds the colour change is compared with the colour chart supplied with the strips.

It has been suggested that every infant should have its urine tested for phenylketonuria during the first weeks of life, and in many areas this is being tried. The objections to such a plan are: (*a*) it is difficult in practice to carry out, (*b*) it is expensive—not a serious argument as even with "phenistix" the cost of testing would be about £6,250 per annum for England and Wales, (*c*) the return would not be worth the time and trouble. If universal testing is not advocated it is important that infants at risk should be tested. They are (1) sibs of known cases of phenylketonuria, (2) sibs of mentally defective children who might be phenylketonuric, and (3) all infants who are to be adopted. Finally, screening for phenylketonuria is essential in assessing any mentally retarded child.

It is probable that most inborn errors of metabolism will be found to follow the pathogenetic pattern described. One gene will be found to influence the chemistry and therefore the activity of one important protein. This is as exciting to us as the discovery that all the varied lesions of syphilis were due to a single causative organism must have been to an older generation. From the primary gene effect flow all the secondary effects consequent upon the deficiency of substances beyond the metabolic block, and excess of normal or abnormal metabolites proximal to it. For instance, there is a rare condition of failure to thrive, jaundice, liver cirrhosis, cataract and mental deficiency, a peculiar set of symptoms and signs, which has now been elucidated. Infants with this trouble, which is familial, excrete galactose in the urine. It is known that the basic lesion is inactivity of the enzyme P-Gal-uridyl-transferase which is necessary for the conversion of galactose to glucose. The apparently unrelated signs of the disease are due either to hypoglycaemia or hypergalactosaemia.

The most common of this rare group of disorders is fibrocystic disease of the pancreas (mucoviscidosis). The primary gene effect is not known, but systems involved include the pancreas (failure of exocrine secretion), the bronchial glands (viscid mucus), and the sweat glands (excess salt). Clinical manifestations vary from neonatal intestinal obstruction (meconium ileus), to intractable pulmonary infection, or steatorrhoea with failure to thrive. It is possible that with the clue of the "one gene, one enzyme or one protein" hypothesis, diseases such as muscular dystrophy may be elucidated. Such diseases cause great anxiety from the genetic viewpoint.

The most common form of muscular dystrophy is the pseudo-hypertrophic type. Unfortunately, its onset is usually in middle childhood so the family may have several children before the condition is revealed. It is usually inherited as a sex-linked recessive, boys being affected and girls transmitting the disease. Of cases at the clinic most have been apparent mutations with a negative family history, but this may be due to selection of cases.

Many problems are posed by genetic diseases of late onset as opposed to conditions apparent at birth. If the parents are aware that a child may be affected with such a disease they will be watchful and anxious for the first signs to appear. This anxiety may affect the medical adviser, on the other hand he may be over-optimistic.

**Mr. and Mrs. C.** are a healthy couple although Mrs. C. has a history of minor mental disease. Their first child died at the age of 3 years from an acute demyelinating disease (Schilder's disease). They were told that this was an inherited condition. Their second child born after the death of the first showed signs of neurological abnormality quite early. This was somewhat masked because the mother was disabled with puerpural psychosis and the child was cared for by relatives. At 1 year she was not sitting up and appeared retarded. There was much anxiety and the parents could not be convinced the child was not about to suffer from the neurological disease which afflicted their first child. The child is now 3 years and has evidence only of a mild hemiplegia, but agony is hardly too strong a word to describe the feelings of the parents while watching the second child develop.

**Mr. and Mrs. M. (S.M.H. 150144)**

The third child of this family died at the age of 23 months, following prolonged convulsions apparently starting with an attack of measles. Measles encephalitis was diagnosed as the cause of death. Their fourth child was healthy, as were the first and second. The fifth child died at the ages of 25 months of convulsions diagnosed as hypsarrhythmia. At this time Mrs. M. was pregnant. The sixth child was apparently normal; however, when he was 18 months old Mrs M. was suspicious that he was going the same way as the previous affected children. Neither family doctor nor paediatrician could find definite signs, and Mrs. M. was heavily reassured. At two years the sixth child was dead, having passed through an exactly similar illness.

**Mr. and Mrs. P. (30808)**

Mr. P. was an apprentice engineer when, at age 18, his shoulders became weak and his facial muscles myopathic. There was a long period of diagnostic argument as to whether his symptoms were due to lead poisoning or to a muscular dystrophy (facio-scapulo-humeral type). When it became clear that he was suffering from muscular dystrophy he had to adjust to a new job and mode of life. This he accomplished, and is now in a responsible clerical post. His son, born in 1942, was watched anxiously and at 16 years showed unmistakable signs of the disease. His adjustment, however, is far less satisfactory and he is bitterly resentful of his inability to play games and to take on any other than clerical work.

It may well be that in certain cases it is inadvisable to enlighten parents concerning the hereditary nature of a disorder as it may provoke, sometimes, needless anxiety; in other cases the anxiety, while justified, may cause maladjustment. Not only the individuals concerned but also the diagnosis and natural history of the disease in question must be considered.

This is an important matter when parents enquire about their children's potential children. Grandparents are often over-anxious and critical, they may be intolerably so if they fear an inherited taint in the grandchildren. One of the most rewarding aspects of genetic counselling is the reassurance that can often be given regarding the children of unaffected members of a family. To reassure justifiably requires considerable knowledge of the interpretation of genetic data.

A group of conditions in which genetic factors are of undoubted importance are the chromosome abnormalities. These seem not to be due to disturbances in the transmission of single genes, but to abnormalities in the chromosome constitution of the gametes during oögenesis or spermatogenesis. Their recognition represents an immensely important scientific advance which has contributed greatly to our knowledge of the aetiology of a relatively common condition, mongolism, and several rare conditions associated with abnormalities of the gonads, Turner's syndrome, Klinefelter's syndrome, and other forms of gonadal agenesis.

Each human cell contains forty-six chromosomes; forty-four autosomes and two sex chromosomes. In the female the sex chromosomes are termed XX and in the male XY. The autosomes are usually numbered. The XX chromosome constitution can be histologically identified owing to the presence of stainable nuclear sex chromatin. This discovery was made by Barr in 1949. Since then, new techniques have made it possible to stain and visualize separately the chromosomes of man (Ford & Hamerton, 1956). Among the earlier rewards of this technique was the demonstration that children with mongolism have forty-five autosomes instead of the normal forty-four. There are now two verified associations with mongolism (*a*) advanced maternal age, and (*b*) trisomy of the chromosomes. It is tempting to relate the two by suggesting that abnormalities of the chromosomes in the gametes are more likely to occur in the "ageing" gonad. There is some evidence that this is so. However, in mongolism there is no conclusive evidence that the chromosomal abnormality is necessarily maternal in origin.

Parents of mongols frequently seek genetic advice. In most cases the mothers are near the menopause and the question of subsequent children is not the important problem. They are more usually seeking an explanation for the misfortune and are worried as to whether their older children may produce mongols. While the new knowledge does not explain the aetiology of mongolism it does enable more information to be given, and the parents can be reassured regarding their children's children. However, some mothers of mongols are young women and it is then more difficult to give a genetic prognosis. Second mongols in a family are very rare but not unknown. It seems reasonable, however, to advise such families not to delay if they are going to have further children, in order to avoid the hazard of advanced maternal age.

It is likely that several more hitherto unexplained conditions may be revealed as due to chromosome abnormalities. So far, the most fruitful area has been in the field of infertility and physical sexual anomalies, multiple congenital malformations, and mongolism. Such cases should receive the benefit of investigation which includes chromosome estimation. The material required is a small piece of skin or bone marrow which is cultured before being prepared for chromosome counting.

### GROUP III. DISEASES WHICH ARE FAMILIAL, BUT NO REGULAR PATTERN OF INHERITANCE IS DISCERNABLE

Thirty-three families were seen. Such common entities as asthma, epilepsy, diabetes mellitus, mental subnormality and congenital malformations of the central nervous system form the majority of cases in this large and somewhat difficult group. They may be divided into two main groups; (*a*) the characteristic is modified by the action of several genes and is continuously distributed through the population and (*b*) the genetic influence is heavily modified by environmental factors.

(*a*) Mental defect is a typical characteristic often falling in this group. In most cases of gross mental defect there is a structural or metabolic cause for the cerebral dysfunction. However, in the lesser degrees of defect the brain is structurally and biochemically normal. These people are merely dull, just as many are short in stature or thin in physique. Intelligence (as defined as that quality measured by "intelligence tests") is a largely inherited characteristic. The dull individual born into a dull home is usually well adapted, but consternation however reigns when a dull individual is produced by bright professional parents.

**Mrs. B.** (60048) had one normal daughter, aged 15, and a mentally defective child of 10 years. The defective child was of normal appearance, had received no brain injury and there was no evidence of a metabolic brain disorder. The family history was studded with dons, writers, and distinguished people in most walks of life. Mr. B. was doing responsible executive work and the possession of a defective child was a severe blow to the parents. Mr. B. was inclined to blame an irresponsible escapade in N. Africa during the war, but one of the distinguished consultants that had seen the child said "it is undoubtedly hereditary and will almost certainly crop up again in the family". If this was meant to console Mr. B. for his night of venery in Alexandria it did so at great cost, for it not only placed a stigma on the family past but filled Mr. and Mrs. B. with fears for the future. In this sort of case we do not know the answer and *primum non nocere* must be the main part of any advice given.

(*b*) Genetic influence modified by environment. In this group come a great many interesting conditions, asthma, some cases of epilepsy, diabetes mellitus, coronary thrombosis, obesity, susceptibility to cancer and tuberculosis and, no doubt, many others. The diseases are undoubtedly familial, but do not follow a regular Mendelian pattern of transmission. Genetic prognosis must be given largely on statistical grounds. Roberts (1959b) has emphasised that from the counselling aspect the more obscure the genetic element the better in general is the genetic prognosis. The application of this principle may be justifiable in reassuring parents but should not lead to the application of less energy in determining the true place of inheritance in these diseases. In order to get the statistical information required large numbers of cases must be studied together with family trees. In some of these conditions advice is sought largely for the sake of the mother, in others for eugenic reasons.

(i) Lethal congenital malformations. About twenty per cent of still births and neonatal deaths are due to congenital malformations. Of these, eighty per cent affect the central nervous system (anencephaly, spina bifida cystica). It is commonly believed that there is a genetic influence in the aetiology of the malformations, as they are more likely to be repeated in a family than can be accounted for by chance. Proof of this is lacking but much more information is required than is currently available about the incidence of these prevalent and distressing conditions. The recurrence rate is low and even after a mother has had two such malformed infants the probability of future normal children is quite good. Family limitation if practised is largely to spare the mother the stress of unsuccessful pregnancy. Genetic advice is limited to giving the statistical chances of recurrence and to stressing the hopeful outlook that is justified.

(ii) Eugenic reasons. Conscientious parents knowing of a condition such as asthma, epilepsy or diabetes in the family may feel that they should not have children if they are likely to be sufferers from these conditions or propagate them. Sometimes the motives behind a consultation are less altruistic.

**Mr. and Mrs. H.** (58896)

Mrs. H. is a young 19-year-old who has suffered for petit mal since the age of 5. She is not seriously handicapped although still on medication. Her parents disapproved of her choice of husband and as part of a campaign of persecution, they said she should not have children because "they were bound to be epileptic" and also suggested that Mrs. H. would not be able to look after them.

It is impossible to give an accurate genetic prognosis in such a case; Mrs. H.'s epilepsy may well be due to birth injury or some other environmental factor, but if genetic the chances of her children being affected are probably small (approximately one in forty), and there are many people prone to epilepsy who have no or few fits. In this case there is, however, the problem of Mrs. H's own fits. The geneticist is not however, concerned with predicting whether a mother will be physically or mentally fit to look after her child. That is another facet of the total problem.

Whether those not physically fit should reproduce is a difficult question, and one which the geneticist would not care to answer in an individual case. Undoubtedly as the treatment of diseases such as diabetes mellitus, haemophilia, asthma and epilepsy improves, more sufferers from these conditions will be able to live normal lives which include reproduction. None can say that an increase in people suffering from these diseases will seriously weaken the human race. They may have qualities which distinguish them from the ordinary in a beneficial sense and thus contribute to rather than detract from the community's welfare. These conclusions apply in some measure to the problems of consaguinity in marriage and to A.I.D.

**Miss R. and Mr. M.** (56532) were proposing to marry. They are first cousins and the family history disclosed: (*a*) menopausal psychosis, (*b*) "mild" form of epilepsy, (*c*) Paget's disease of bone, and (*d*) mental defect.

None of these conditions is known to be due to recessive genes and there was no reason to interdict the marriage. Degrees of consanguinity other than incest and first cousin matings are probably quite unimportant. Incestuous matings are likely to manifest undesirable recessive characteristics. First cousin marriage increases the relative risk from recessive genes, but the actual risk is not considerable. There is some evidence that the offspring of first cousin marriages tend to be infertile. Problems of A.I.D. have not as yet appeared at the genetic clinic. The major genetic hazard of A.I.D. would seem to be a remote possibility of a person marrying his or her half-sibling. Such an occurrence would be extremely rare and not necessarily harmful. Obviously, a donor of spermatozoa should be chosen who is free from obvious inherited disease, and in this respect the child born by A.I.D. may have a superior genetic endowment from his "selected" father than from one chosen by chance!

In our experience genetic advice is welcomed and usually we find that the genetic prognosis is better than the patients had thought possible. It is likely that many parents would appreciate genetic advice but do not like to ask, or do not realise that it is available. The accuracy and value of such advice has been greatly increased by the new facts of genetics, and human genetics has a considerable part to play in the advancement of scientific knowledge.

### ACKNOWLEDGEMENTS

My thanks are formally due to Dr. J. A. Fraser Roberts for his permission to use the material from the Bristol Genetic Clinic. My debt to him is immeasurably greater than this and he is responsible for encouragement, stimulation and guidance for the whole of the period covered by this review. He has also read and constructively criticized the manuscript. In the preparation of this paper, Miss Belinda Davies, a Paediatric Clerk at the time, gave invaluable help and enthusiasm.

### REFERENCES

Barr, M. L., Bertram, E. G. (1949). *Nature* (Lond.) **163,** 676.
Ford, C. E., Hamerton, J. L. (1956). Ibid, **183,** 302.
Hsia, D. Y. (1959). *The Inborn Errors of Metabolism.* The Year Book Publishers Inc. Chicago.
Nance, W. E. (1959). *Medicine* (Baltimore). **38,** 403.
Roberts, J. A. F. (1959a). *Brit. Med. Bull.* **15,** 129; (1959b). *An Introduction to Medical Genetics.* 2nd. edition. Oxford University Press. London.

